# Fucoxanthin and Its Metabolite Fucoxanthinol in Cancer Prevention and Treatment

**DOI:** 10.3390/md13084784

**Published:** 2015-07-31

**Authors:** Luc J. Martin

**Affiliations:** Biology Department, Université de Moncton, Moncton, NB E1A 3E9, Canada; E-Mail: Luc.Martin@umoncton.ca; Tel.: +1-506-858-4937; Fax: +1-506-858-4541

**Keywords:** fucoxanthin, fucoxanthinol, viability, apoptosis, cancer

## Abstract

Fucoxanthin is a carotenoid present in the chloroplasts of brown seaweeds. When ingested, it is metabolized mainly to fucoxanthinol by digestive enzymes of the gastrointestinal tract. These compounds have been shown to have many beneficial health effects, including anti-mutagenic, anti-diabetic, anti-obesity, anti-inflammatory and anti-neoplastic actions. In every cancer tested, modulatory actions of fucoxanthinol on viability, cell-cycle arrest, apoptosis and members of the NF-κB pathway were more pronounced than that of fucoxanthin. Anti-proliferative and cancer preventing influences of fucoxanthin and fucoxanthinol are mediated through different signalling pathways, including the caspases, Bcl-2 proteins, MAPK, PI3K/Akt, JAK/STAT, AP-1, GADD45, and several other molecules that are involved in cell cycle arrest, apoptosis, anti-angiogenesis or inhibition of metastasis. In this review, we address the mechanisms of action of fucoxanthin and fucoxanthinol according to different types of cancers. Current findings suggest that these compounds could be effective for treatment and/or prevention of cancer development and aggressiveness.

## 1. Introduction

Fucoxanthin (Fx) is a naturally occurring brown- or orange-coloured pigment that belongs to the class of non-provitamin A carotenoids present in the chloroplasts of brown seaweeds. It is the most abundant of all carotenoids, accounting for more than 10% of the estimated total natural production of carotenoids [1]. It forms a complex with chlorophyll–protein and plays an important role in light harvesting and photoprotection for effective light use and up-regulation of photosynthesis. Under experimental conditions in mice, oral administration of Fx does not exhibit toxicity and mutagenicity [2,3].

### Sources and Metabolites of Fucoxanthin

**Figure 1:**
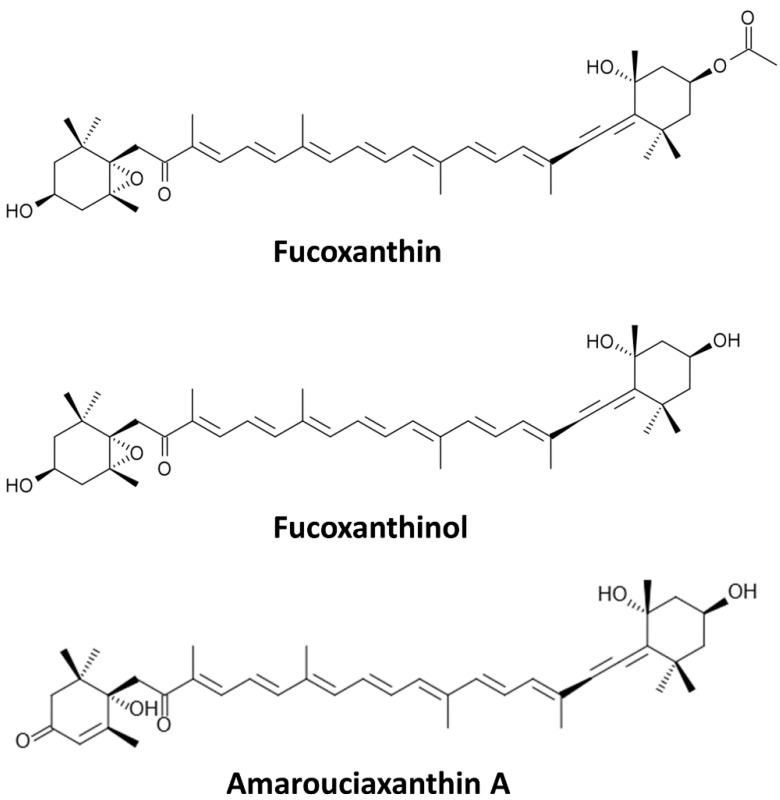
Chemical structures of Fucoxanthin (Fx) and of its metabolites Fucoxanthinol (Fxol) and Amarouciaxanthin A.

Fx (Figure 1) is a predominant carotenoid found in edible brown algae, such as wakame (*Undaria pinnatifida*), kombu (*Laminaria japonica*), hijiki (*Hijikia fusiformis*), arame (*Eisena bicyclis*) and *Sargassum fulvellum* [4,5]. Carotenoids and Fx, being hydrophobic, are absorbed at the intestinal level through the same path as dietary fats. Ingested Fx is metabolized mainly to fucoxanthinol (Fxol), which is further converted to amarouciaxanthin A in the liver [3,6]. Dietary Fx is hydrolysed to Fxol in the gastrointestinal tract by digestive enzymes, such as lipase and cholesterol esterase, and then absorbed into intestinal cells [7]. Thus, the bioactive forms of Fx *in vivo* are Fxol and/or amarouciaxanthin A. However, limited research covers the actions of amarouciaxanthin A in cancer cells. Nonetheless, Fx and Fxol have many beneficial health effects, including anti-mutagenic [8], anti-diabetic [9], anti-obesity [10], anti-inflammatory [11,12] and preventive actions on liver, breast, prostate, colon and lung cancers [8,13,14,15,16,17]. In this review, cancer prevention using Fx and its metabolite Fxol, as well as their possible mechanisms of action, will be discussed according to different types of cancers.

## 2. Cancer Prevention

Fx and Fxol exert their anti-proliferative and cancer preventive influences via different molecules and pathways involved in either cell cycle arrest, apoptosis, or metastasis (reviewed in [18]). In addition, Fx has been shown to have anti-angiogenic potential using human umbilical vein endothelial (HUVEC) cells [19], thus, contributing to cancer prevention. Indeed, Fx suppresses the mRNA expression of pro-angiogenic fibroblast growth factor 2 (FGF-2) and its receptor (FGFR-1) as well as their trans-activation factor, early growth response protein 1 (EGR-1), known to activate FGF-2 transcription [20]. In addition, Fx also resulted in down-regulation of the phosphorylation of FGF-2-mediated by intracellular signalling proteins, such as extracellular signal-regulated kinases (ERK1/2) and protein kinase B (Akt) [19], leading to reduced migration of endothelial cells. Fx has also been shown to inhibit cancer cells proliferation by increasing gap junction intercellular communication in human cancer cells [21], possibly leading to increased intracellular signalling promoting cell cycle arrest and apoptosis.

Having low toxicity for normal cells, Fx and its metabolites show great promises as chemopreventive and/or chemotherapeutic agents in cancer. In the next sections, the anticancer effects of Fx and Fxol will be reported according to cancer types.

### 2.1. Osteosarcoma

Chances of survival of osteosarcoma patients depend on prevention and treatments of metastasis. Anti-osteosarcoma properties of several carotenoids have been evaluated in cell lines. Among them, Fx and, to a greater extent, Fxol inhibited cell viabilities of Saos-2, LM8, MNNG and 143B cells [22]. Specifically, Fxol induced cell cycle arrest by reducing the expression of cyclin-dependent kinase 4 (CDK4), cyclin-dependent kinase 6 (CDK6) and cyclin E, and increased apoptosis by reducing the expressions of important anti-apoptotic mediators such as survivin, XIAP, Bcl-2 and Bcl-xL and increasing activation of caspases-3, -8 and -9 [22]. Fx treatments also inhibited the expressions of proliferative mediators, such as survivin, vascular endothelial growth factor (VEGF) and epidermal growth factor receptor (EGFR) [23].

Cell growth and survival of osteosarcoma cells is also dependent on the activity of the phosphatidylinositol 3-kinase (PI3K)-Akt pathway [24]. Akt prevents apoptosis through phosphorylation of caspase-9 and the cell cycle regulator glycogen synthase kinase-3β (GSK3β), as well as activation of NF-κB transcription factors [25,26]. Interestingly, Fxol resulted in inhibition of phosphoinositide-dependent kinase 1 (PDPK1) phosphorylation, leading to reduced phosphorylations of Akt and of its target GSK3β [22]. Being a downstream target of GSK3β, the proto-oncogene β-Catenin was also downregulated by Fxol in Saos-2 cells [22]. β-Catenin is known to regulate cell-to-cell adhesion and its mutations or overexpression are associated with the development of many types of cancers, including osteosarcoma [27,28].

Cell migration and invasiveness of osteosarcoma cells is known to be promoted by the upregulation of the expression of matrix metalloproteinase-1 (MMP-1) through activation of activator protein-1 (AP-1) transcription factors [29]. MMP-1 expression was inhibited by Fxol through reduction of AP-1 signalling in Saos-2 cells [22]. Thus, Fxol showed promising anti-metastatic activities on osteosarcoma cells by blocking AP-1, resulting in inhibition of MMP-1.

Normally, signal transducers and activators of transcription (STAT)-3/5 transcription factors contribute to tumourigenesis when deregulated [30,31]. In sarcoma cells, high concentrations of Fx (50–150 µM) has been shown to inhibit STAT3 expression and phosphorylation [23]. Anti-tumour effects of Fx have also been investigated on xenografted sarcoma 180 in mice, resulting in significant growth inhibition with increased apoptosis as a result of epidermal growth factor receptor (EGFR)/Janus kinase (JAK)/STAT signalling disruption [23]. Therefore, Fx, and possibly Fxol, may prevent sarcoma development through modulation of numerous signalling pathways involved in tumourigenesis and metastasis.

### 2.2. Leukemia and Lymphoma

Hematologic cancers are the most common malignancies of childhood. Leukemia is characterized by an increased cell division of white blood cells, leading to impairment in the maintenance of red blood cells population. Unlike leukemia, lymphoma specifically affects the lymph nodes. Fx and Fxol have promising inhibitory effects on proliferation of these cancers. Indeed, these compounds have been shown to trigger apoptosis through induced procaspase-3, -7, and poly (ADP-ribose) polymerase (PARP) cleavages and through regulation of Bcl expression (Bcl-2 and Bcl-xL) in human leukemia (HL-60) cancer cells [32,33]. However, others have shown that Fx treatment of these cells increased cleavages of procaspase-3 and PARP without any effect on the protein levels of anti-apoptotic Bcl-2, Bcl-xL, or pro-apoptotic Bax [34].

In primary effusion lymphoma cells, a rare and highly aggressive non-Hodgkin’s lymphoma caused by human herpesvirus 8, Fxol-induced suppression of cell viability was more pronounced than that of Fx [35]. Fx and Fxol also induced cell cycle arrest in G0/G1 phase, increased caspase-dependent apoptosis, inhibited the activation of NF-κB, AP-1 and Akt, and down-regulated anti-apoptotic proteins (Bcl-xL and XIAP) in these cells [35]. Consistent with these results, Fx has also been shown to inhibit the nuclear translocation of p50 and p65 proteins in lipopolysaccharide (LPS)-stimulated RAW 264.7 macrophages, resulting in lower levels of nuclear transactivation by NF-κB transcription factors [11,36].

Adult T-cell leukemia (ATL) is a fatal malignancy of T lymphocytes caused by human T-cell leukemia virus type 1 (HTLV-1) infection and remains incurable. Interestingly, Fx and Fxol inhibited cell viability of HTLV-1-infected T-cell lines and ATL cells, with a higher potency of Fxol compared to Fx [37]. Both carotenoids induced cell cycle arrest during G0/G1 phase by reducing the expression of cyclin D1, cyclin D2, CDK4 and CDK6, and inducing the expression of Growth Arrest and DNA Damage 45 alpha (GADD45α) [37]. Being involved in DNA repair, cell cycle control, senescence, genotoxic stress and having pro-apoptotic activities, GADD45α is known to have multiple inhibitory effects on tumourigenesis [38]. Fx and Fxol also induced apoptosis of ATL cells by reducing the expressions of anti-apoptotic Bcl-2, XIAP, cIAP2 and survivin and activation of procaspase-3, -8 and -9 [37]. Fx and Fxol suppressed IκBα phosphorylation and JunD expression, resulting in inactivations of NF-κB and AP-1 [37]. Confirming these results, mice with severe combined immunodeficiency and harboring tumours induced by inoculation of HTLV-1-infected T cells responded to treatment with Fxol by suppression of tumour growth [37], thus suggesting that Fx and Fxol could be useful therapeutic agents for patients with ATL.

Effects of Fx and Fxol on B-cell malignancies, such as Burkitt’s lymphoma, Hodgkin’s lymphoma and Epstein-Barr virus-immortalized B cells have been investigated. Fx and, with a higher efficiency, Fxol, reduced viability of these malignant B cells in a dose-dependent manner with increased cell cycle arrest during G0/G1 phase and caspase-dependent apoptosis [39]. In these cells, Fxol inhibited NF-κB activity, leading to downregulation of NF-κB-dependent anti-apoptotic and cell cycle regulator genes, such as Bcl-2, cIAP-2, XIAP, cyclin D1 and cyclin D2 [39]. Accordingly, Fx and Fxol could be considered as anti-proliferative or preventive agents against leukemia and lymphomas.

### 2.3. Lung Cancer

Lung cancer is one of the most common cancers worldwide and is still a leading cause of cancer death in North America. Although carotenoid levels are well known to be associated with a lower risk of lung cancer death [40], inhibitory actions of Fx and Fxol on this type of cancer have been barely investigated. However, in experimental lung metastasis *in vivo* assays, Fx resulted in reduced tumour nodules formation [41]. In addition, extracts from New Zealand seaweed *Undaria pinnatifida* containing low levels of Fx were found to have inhibitory effects on growth of human lung carcinoma cell lines A549 and NCI-H522 [42]. Thus, more studies using Fx and/or Fxol as preventive agents against lung cancer metastasis may be promising to lower the important death rate associated with such complications.

### 2.4. Prostate Cancer

With the increased life expectancy, it is anticipated that the diagnosis of prostate cancer will increase by 55% between 2010 and 2030 [43]. Fx has been shown to reduce viability and induce apoptosis in human prostate cancer cells (PC-3, DU 145 and LNCaP) [44]. As in HepG2 liver cancer cells, Fx was also shown to induce cell cycle arrest in G1 and increase GADD45 gene expression in DU145 and LNCaP cells, possibly through activation of c-Jun *N*-terminal kinase (SAPK/JNK) [45,46,47]. This subfamily member of the mitogen-activated protein kinases (MAPKs) is known to be an upstream activator of GADD45 expression [48]. In addition, Fx has been shown to induce procaspase-3 and PARP cleavages, reduce the expression of anti-apoptotic Blc-2 and, surprisingly, of pro-apoptotic Bax proteins, leading to increased apoptosis of human prostate cancer PC-3 cells [17]. Interestingly, Fx was converted to Fxol within treated PC-3 cells [17]. Compared to Fx, Fxol had a stronger anti-proliferative effect on PC-3 cells [6]. Hence, effects of Fx on prostate cancer cells apoptosis may rather be attributed to Fxol and such conversion of Fx to Fxol is normally limited to the gastrointestinal tract.

### 2.5. Gastrointestinal Cancer

With recent changes in nutritional habits of Asians, gastrointestinal cancer incidence is increasing at an alarming rate in this population [49]. As in other cancers, Fx has been shown to suppress the level of Bcl-2 protein and induce apoptosis in human colon cancer cells (Caco-2, HT-29 and DLD-1) [50]. In addition, Fxol also had a stronger anti-proliferative effect than Fx on Caco-2 human colon cancer cells [33].

Cell proliferation may be associated with retinoblastoma protein (Rp) phosphorylation, releasing E2F transcription factors and promoting G1 to S phase transition [51,52]. In WiDr human colon adenocarcinoma cells, Fx may induce cell cycle arrest during the G0/G1 phase and apoptosis through inhibition of Rb phosphorylation and increased the expression of a CDK and proliferating cell nuclear antigen (PCNA) inhibitory protein, p21WAF1/Cip1 [16]. As in sarcoma, high concentrations of Fx (50–150 µM) also inhibited STAT3 expression and phosphorylation, resulting in downregulation of cyclin B1 in gastric adenocarcinoma cells [53].

Interestingly, Fx has been shown to reverse multidrug (such as 5-FU, vinblastine and etoposide) resistance of Caco-2 cells by interfering with ATP-binding cassette (ABC) transporters [54], an important strategy to overcome multidrug resistance in cancer pharmacotherapy [55]. Thus, Fx may be used to sensitize colon cancer cells and possibly other types of cancers to chemotherapeutic drugs.

### 2.6. Liver Cancer

Liver cancer is one of the most serious types of cancer with a five year survival rate of 14% after diagnosis [56]. Fx has been shown to significantly inhibit proliferation of human hepatoma HepG2 cells by 28% after 48 h of incubation with a 10 μM dose [57]. Growth inhibition of HepG2 cells by Fx was triggered by induction of cell cycle arrest in the G0/G1 phase, down-regulation of cyclin D/CDK4 complex and decreased phosphorylation of the retinoblastoma protein (Rb) [58]. In addition, others have also shown that Fx treatments resulted in inhibition of p38 MAPK and increased GADD45α expression [45]. Interestingly, Fx also inhibited proliferation of SK-Hep-1 human hepatoma cells through G1 cell cycle arrest and increased apoptosis, whereas murine embryonic hepatic (BNL CL.2) cells growth was enhanced [21]. Thus, antiproliferative actions of Fx were selective to liver cancer cells compared to normal embryonic hepatic cells.

These results may be explained, in part, by the influence of Fx on cell-to-cell communication. Indeed, Fx enhanced gap junctional intercellular communication of SK-Hep-1 cells by increasing connexin43 and connexin32 expressions, but not of mouse liver BNL CL.2 cells [21]. Increased cell-to-cell communication may result in modulation of cytosolic calcium levels, contributing to cell cycle arrest and apoptosis.

Drug resistance is a major inconvenient lowering the efficacy of chemotherapy. The Pregnane X receptor (PXR) is known to regulate the expression of drug-metabolizing enzymes, such as the cytochrome P450 3A (CYP3A) family, and transporters, like multiple drug resistance 1 (MDR1), thus promoting drug resistance [59]. In addition, PXR activation was found to increase tumour aggressiveness of primary human colon cancer tissue xenografted into immunodeficient mice [60]. Interestingly, Fx has been shown to attenuate rifampicin-induced CYP3A4 and MDR1 gene expressions through inhibition of PXR interaction with the steroid receptor coactivator-1 (SRC-1) in HepG2 cells [61]. Moreover, when combined with the anticancer drug cisplatin, Fx further decreased HepG2 cells proliferation [57]. To do this, Fx inhibited cisplatin-induced expressions of NF-κB, excision repair cross complementation 1 (ERCC1) and thymidine phosphorylase (TP) [57], leading to improvement of chemotherapeutic efficacy of cisplatin by activation of apoptosis, inhibition of DNA repair and promoting tumour regression. As in gastrointestinal cancer, Fx may also be used to sensitize liver cancer, and possibly other types of cancers, to chemotherapeutic treatments.

Although numerous effects of Fx on liver cancer have been reported, it is important to recall that dietary Fx is hydrolysed into Fxol in the gastrointestinal tract before absorption in the intestine. Then, hepatocytes and HepG2 are able to convert Fxol into amarouciaxanthin A [6], suggesting that the latter compound may be responsible, in part, for the anti-proliferative effects of Fx in liver cancer.

### 2.7. Bladder Cancer

Urinary bladder cancer development, influenced by genetic and environmental factors, often results into multiple tumours appearing at different times and different locations within the bladder. Fx reduced viability and triggered apoptosis of human bladder cancer EJ-1 cells as a result of caspase-3 activation [62]. Fx also inhibited human T24 bladder cancer cells proliferation in a dose- and time-dependent manner through growth arrest at G0/G1 phase of cell cycle, which is mediated by the up-regulation of p21, a cyclin-dependent kinase (CDK)-inhibitory protein and the down-regulation of CDK-2, CDK-4, cyclin D1, and cyclin E [63]. In addition, high levels of Fx induced apoptosis of T24 cells by the abrogation of mitochondrial mortalin-p53 complex, translocation and transcriptional activation of p53 [63]. Mortalin is a stress regulator and a member of heat shock protein 70 known to induce cell differentiation. Thus, these fragmentary results suggest that Fx, and possibly Fxol, may act as chemopreventive agents against bladder cancer development.

### 2.8. Skin Cancer

Since 1970, skin cancer had the second highest increase in mortality rate in Canada [64]. Fx has been shown to inhibit metastatic potential of melanoma cancer cells [41]. Metastasis is a major determinant of mortality in malignant cancers. Fx inhibited the expression and secretion of matrix metalloproteinase-9 (MMP-9), linked to tumour invasion and migration, and also suppressed invasion of highly metastatic B16-F10 melanoma cells [41]. In addition, Fx also reduced the expressions of cell surface glycoprotein CD44 and CXC chemokine receptor-4 (CXCR4), known to be involved in migration, invasion and cancer-endothelial cell adhesion [41]. Hence, the inhibitory effects of Fx on melanoma cells’ adhesion to endothelial cells strongly support its anti-metastatic potential.

In addition, Fx caused cell cycle arrest of B16-F10 cells through decreased protein expressions of phosphorylated-Rb, cyclin D (1 and 2) and CDK4 as well as up-regulation of protein levels for p15(INK4B) and p27(Kip1), which are important cyclin-dependent kinase inhibitors involved in repression of tumourigenesis [65]. Fx also induced apoptosis of these cells through down-regulation of Bcl-xL, resulting in activations of caspase-9, caspase-3, and PARP [65].

In melanoma and UVB-induced skin pigmentation, Fx inhibited tyrosinase activity and melanogenesis, whereas topical applications suppressed cyclooxygenase (COX)-2, endothelin receptor A, p75 neurotrophin receptor (NTR), prostaglandin E receptor 1 (EP1), melanocortin 1 receptor (MC1R) and tyrosinase-related protein 1 (Tyrp1) mRNA expressions [66]. These are all stimulants of melanogenesis of epithelial cells. Such inhibitory effects of Fx on melanogenesis may be attributed to suppression of prostaglandin E2 (PGE2) synthesis and of melanocyte-stimulating hormone receptor (MC1R), resulting in reduced phospholipase C and PKA signalling and leading to suppression of Tyrp1 expression and melanin formation. Altogether, these results suggest that Fx may also be used as an anti-pigmentary ingredient for cosmetics.

### 2.9. Cervical Cancer

Cervical cancer arises from malignant cells originating from the cervix and is among the most prevalent cancers in women. The enzyme phosphatidylinositol 3-kinase (PI3K) is known to be overexpressed in cervical cancer, leading to phosphorylation of Akt and activation of mTOR signalling. Interestingly, Fx has been shown to induce apoptosis in human cervical cancer HeLa cells through inhibition of the PI3K/Akt/mTOR pathway [67,68]. Fx also increased expressions of important mediators of autophagy, LC3 II and Beclin 1 [67], potentially leading to non-apoptotic cell death. Accordingly, Fx and possibly Fxol could be used as a preventive agent against cervical cancer in highly predisposed women.

### 2.10. Breast Cancer

Breast cancer is the most common cancer diagnosed in women worldwide. Several studies have linked diets rich in carotenoids with reduced risk of chronic diseases and cancers, including breast cancer [69,70,71]. Indeed, Fx has been shown to be highly cytotoxic to MCF-7 cells [72]. In addition, our research group have shown that 10–20 µM Fx and Fxol have inhibitory effects on cell viability and apoptosis-inducing effects on two human breast cancer cell lines, MCF-7 and MDA-MB-231 [73,74]. Fxol induced apoptosis of breast cancer cells MDA-MB-231 and MCF-7 by increased cleavages of procaspase-3 and PARP [33,73].

In our research, we looked at possible differences in the inhibitory actions of Fx and Fxol on components of the NF-κB pathway between estrogen sensitive MCF-7 and estrogen resistant MDA-MB-231 breast cancer cell lines. Estrogen receptors play an important role in breast cancer; women with ER positive tumours have an overall better prognosis and are more likely to have their tumours respond to therapy. However, in up to 25% of cases, ER positive tumours are non-responsive to therapy as a result of acquired resistance, possibly linked to constitutive NF-κB leading to estrogen-independent growth [75,76,77]. Indeed, constitutive nuclear localization of p50, p52, c-Rel, and overexpression of p100/p52 in breast cancer have been reported [78,79]. Among NF-κB members, p65 and p50 are constitutively active and overexpressed in breast cancer cells [80], resulting in increased transcription of anti-apoptotic genes [81]. Major differences were observed in the inhibitory mechanisms between Fx and Fxol on members of the NF-κB pathway in breast cancer cell lines. Interestingly, the apoptosis-inducing activities of Fxol were more potent than that of Fx and were correlated, for hormone independent MDA-MB-231 cells, to inhibitory actions on members of the NF-κB pathway p65, p50, RelB, and p52/p100 [73,74]. Thus, enhanced sensitivity of breast tumour cells to apoptosis in response to Fx/Fxol may be associated in part with inhibitory actions on the NF-κB pathway [82,83]. Fxol treatment may contribute to reduce viability of aggressive estrogen-independent tumour growth and may involve inhibitions of nuclear translocation and transcriptional activity of members of the NF-κB pathway. However, more research will be required to clearly establish a regulatory action of Fxol on members of NF-κB.

Being overexpressed and regulated by NF-κB in different types of cancers [84,85,86], SOX9 expression is highly correlated to invasiveness and poor clinical outcome of breast cancer. Indeed, cytoplasmic accumulation of SOX9 has been shown to increase in invasive ductal carcinoma and metastatic breast cancer [87]. The transcription factor SOX9 have been identified as a downstream target of different signalling pathways contributing to breast cancer aggressiveness [88,89,90,91,92,93]. In MDA-MB-231, SOX9 was decreased at the nuclear level by Fx and Fxol [73]. Hence, SOX9 may be involved in MDA-MB-231 cells proliferation and downregulation of its expression and/or phosphorylation may contribute to the inhibitory effects of Fx and Fxol on viability of estrogen resistant breast cancers.

## 3. Conclusions

**Figure 2:**
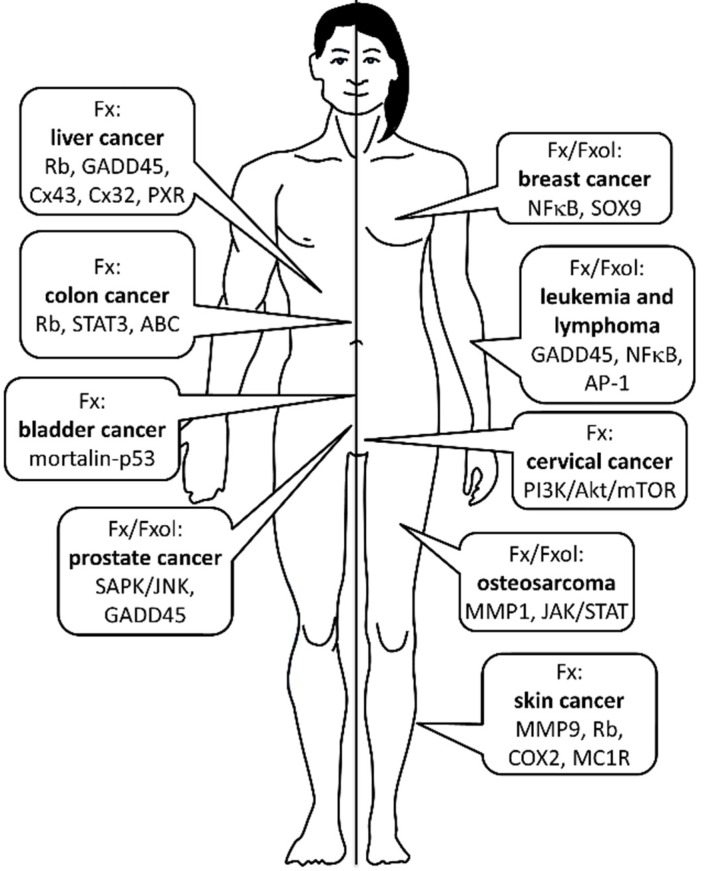
Anticancer effects of Fucoxanthin (Fx) and Fucoxanthinol (Fxol) according to different types of cancers. Most important molecular pathways involved in Fx/Fxol’s actions are also depicted. No sex differences have been made regarding the efficiency Fx/Fxol against any types of cancers. See text for more details.

As summarized in Figure 2, anticancer effects of Fx and Fxol are mediated through several mechanisms including inhibition of cell proliferation, induction of apoptosis, cell cycle arrest and anti-angiogenesis. In addition, combined treatments of Fx or Fxol with anticancer drugs may lead to important new therapeutic strategies having limited multi-drug resistance against many types of cancers. According to current results, Fx and Fxol can induce growth inhibition and apoptosis in different cancer cell types; however, the effective concentrations differ among cancers, and the detailed molecular mechanisms remain to be elucidated. Most dietary Fx may be converted to Fxol, and the latter may exert a suppressive effect on cancer cells more efficiently than Fx *in vivo*. Taken together, current findings suggest that Fxol and Fx could be potentially effective for the treatment and/or prevention of different types of cancers.
